# Historical and social science perspectives on food allergy

**DOI:** 10.1111/cea.14360

**Published:** 2023-06-12

**Authors:** Matthew Smith

**Affiliations:** ^1^ University of Strathclyde Glasgow UK

**Keywords:** anthropology, food allergy, history, immunology, sociology

## Abstract

This article provides an overview of the insights social scientists, historians and other health humanities scholars have made to our understanding of food allergies. It shows how humanities and social science scholars have tended to address three pivotal issues related to food allergies: first, they have addressed the epidemiology of food allergies, including the apparent rise in the rate of food allergies and the emergence of theories that purport to explain why food allergies may be increasing. These include theories related to changes in food consumption and the hygiene hypothesis. Second, humanities and social science scholars have researched how risks related to food allergies have been constructed, understood, experienced and mitigated. Third, humanities and social science scholars have investigated the experiences of food allergy sufferers and those who care for them, providing valuable qualitative insights that can inform how we respond to food allergy and our understanding of the aetiology of food allergy. The article concludes with three recommendations. First, there should be a more interdisciplinary approach to food allergy research that involves social scientists and health humanities scholars. Second, humanities and social sciences scholars should be more willing to unpack and scrutinise the theories put forward to explain the aetiology of food allergies, rather than accepting them at face value. And finally, humanities and social sciences scholars can play a major role in ensuring that the experiences of patients and their carers are articulated and fed into debates about food allergy, including its causes and how to respond to it.

## Graphical abstract

Historians and social scientists scholars have addressed three issues related to food allergies: First, they have addressed epidemiology, including the apparent rise in the rate of food allergies and the theories proposed to explain these increases. Second, they have explored how risks related to food allergies have been constructed, understood, experienced and mitigated. Third, they have investigated the experiences of food allergy sufferers, providing qualitative insights that can deepen our understanding of food allergy.

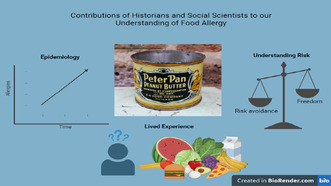

**Key messages**

- Humanities and social science research can inform our understanding of food allergy.
- Scholars have concentrated on the epidemiology, risks posed by and experiences of food allergy.
- Humanities and social science scholars should play a larger role in food allergy research.

## 1 INTRODUCTION

The sudden, unexpected deaths of young people such as Natasha Ednan‐Laperouse from food anaphylaxis, along with tightening allergy labelling legislation and increased service provision, has led to significant and increasing media attention on food allergy.^1^ Since the 1990s, food allergy fatalities have attracted considerable media coverage.^2^ What has often been overlooked in the coverage of these tragedies, however, is wider questions about why food allergies are thought to be increasing, why they are so controversial, how the science of food allergy developed and what food allergies represent about society more generally.

Humanities and social science scholars can help answer these questions, playing a vital role in informing responses to food allergies. In what follows, the contributions of humanities and social science scholars are broken up into three themes, specifically: (1) explaining the rise of food allergies, (2) assessing public health responses to food allergies and (3) patient and parent experiences of food allergies. The article concludes with some recommendations for future research and how humanities and social science insights can best inform clinical practice and allergy research.

## 2 ARE FOOD ALLERGIES RISING AND, IF SO, WHY?

Food allergies have become more widely recognised in many societies since the 1990s. More people appear to have food allergies today and there is greater awareness of them, thanks in part to public health campaigns. In many countries, most restaurants and cafes identify allergens, most supermarkets have ‘free‐from’ sections and most schools and nurseries are ‘nut‐free’ zones. Nevertheless, debates continue about how much of the perceived increases in food allergy are real or merely a reflection of increased awareness.^3^, ^4^ Historical research can provide a longer perspective.

**FIGURE 1:**
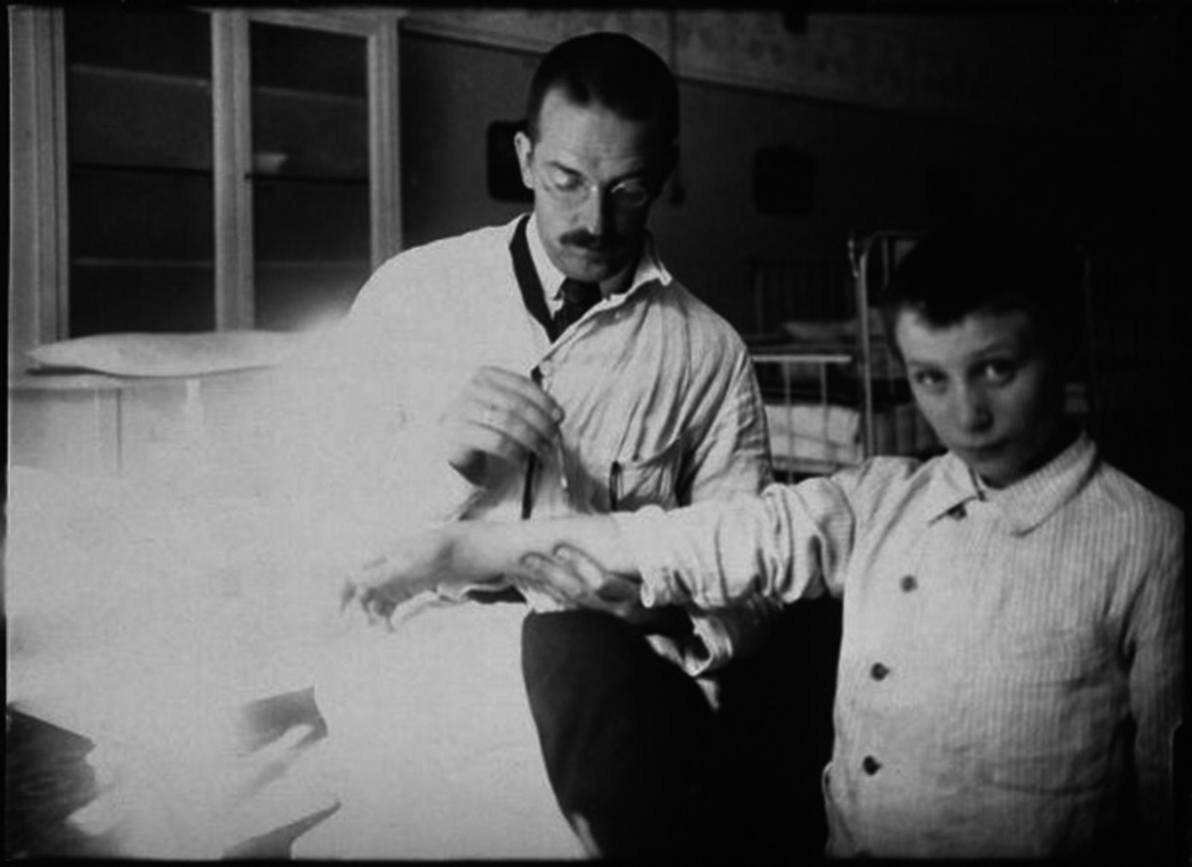
Clemens von Pirquet and a patient. https://wellcomecollection.org/works/yd78ffbn.

The term ‘allergy’ was coined by Austrian paediatrician Clemens von Pirquet (1874–1929) in 1906 as ‘any form of altered biological reactivity’, a broad definition that precipitated arguments about how to define food allergy and distinguish it from food intolerance.^5^ Until the 1930s, however, the term anaphylaxis, coined 4 years earlier by French Nobel laureate, Charles Richet (1850–1935), was the catch‐all for dysfunctional immune responses to insect stings, pollen and food.^6^ The term anaphylaxis later took on its current definition of a severe, life‐threatening allergic reaction (Figure 1).

**FIGURE 2:**
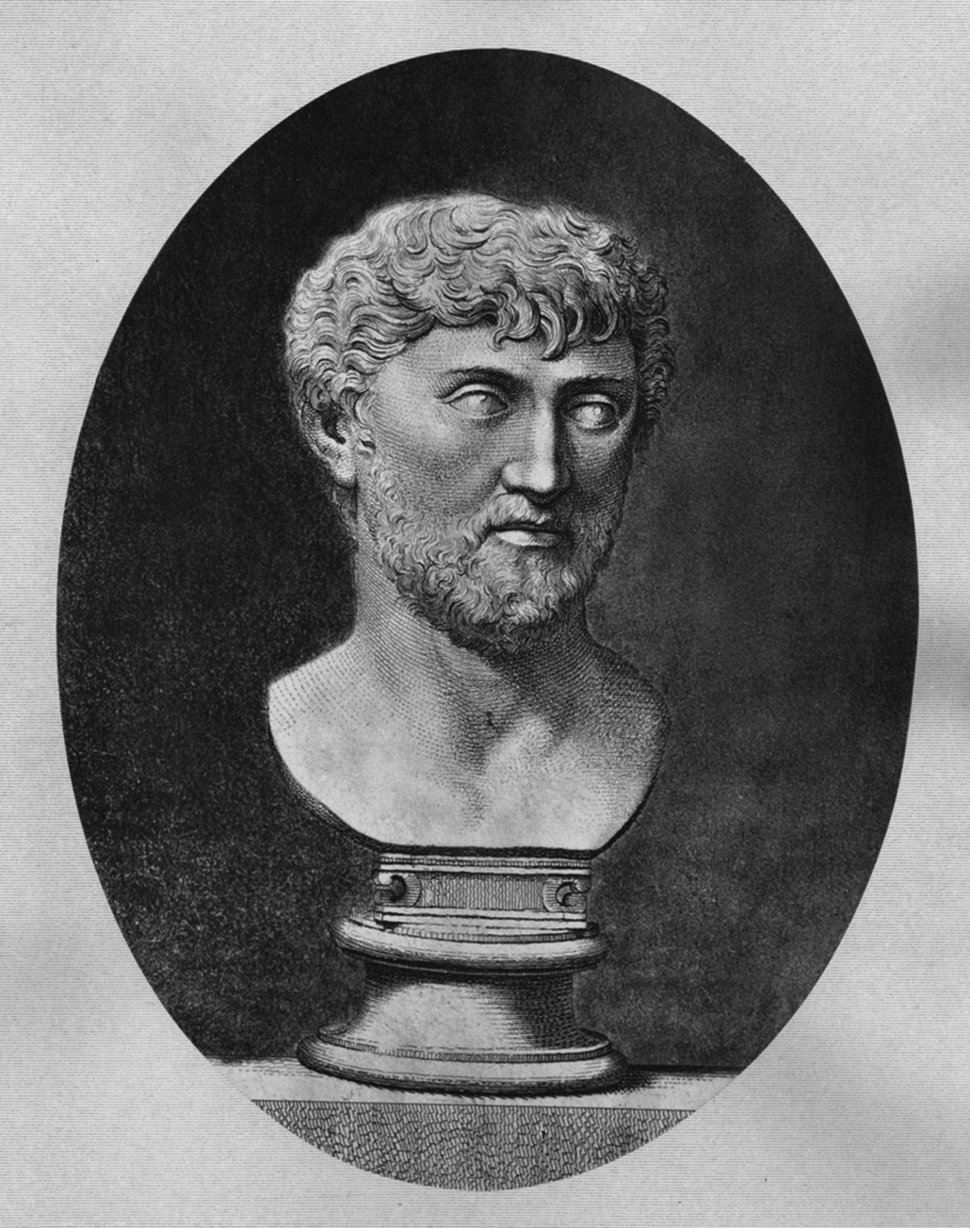
Lucretius (99–55 BCE). https://en.wikipedia.org/wiki/Lucretius#/media/File:Lucretius1.png.

What about strange reactions to foods observed prior to the twentieth century? Historians get irked when today's diagnostic terms are carelessly applied to historical figures.^7^ This is particularly problematic with psychiatric diagnoses, which vary enormously across time and place,^8^ but quite speculative retrospective diagnosis of allergy also occurs.^9^ With food allergies, however, we are assisted by the fact that the term idiosyncrasy was often used to describe unusual reactions to food. According to Jackson, the most common idiosyncrasies identified since the time of Hippocrates were related to food. (Table 1)^5^ It is also possible to identify cases where physicians blame familiar symptoms, such as eczema, urticaria, migraine and asthma, on food.^2^ This does not definitively mean that such cases were caused by food allergies. But it does indicate that for at least 2500 years, people have recognised that certain people react pathologically to foods that others find perfectly healthy. Indeed, historical records suggest the possibility that advice to the Chinese emperors Shen Nong and Huang Di over 4500 years ago included avoidance of specific foods during pregnancy, and to manage skin conditions which might have included eczema.^10^ The concept of idiosyncratic reactions to foods, or what we would today call food hypersensitivity, is clearly recognised in the Roman philosopher‐poet Lucretius's (98–55 BC) declaration that, ‘food to one, is poison to another’. Strange reactions to foods have also been described by Ibn Sina (980–1037), Sir Thomas More (1478–1535), Sir John Floyer (1649–1734) and Henry Hyde Salter (1823–1871), among others (Figure 2).^11^

**TABLE 1:** Food allergy timeline.

c. 2700 BCE	Written advice to Chinese emperors Sheng Nong and Huang Di includes that pregnant women should avoid eating shrimp, chicken, and meat and that specific foods should be avoided for people with skin ‘ulcerations’
c. 450–400 BCE	Hippocrates (460–370 BCE) observes that some people react strangely after eating cheese
c. 100 BCE	Mithridates (135–63 BCE), the rule of Pontus in Anatolia, is said to develop an immunity to poisons by ingesting micro‐doses, referred to as mithridatism, and seen as a precursor to desensitisation therapy
c. 55 BCE	Lucretius (98–55 BCE) declares that ‘food to one, is poison to another’
c. 1000	Persian physician Ibn Sina (980–1037) discusses ‘idiosyncrasies’ to foods, stating that ‘good and laudable foods may be injurious to some’
c. 1200	Moses Maimonides (1135–1204) describes how certain foods can trigger asthma
1513–1518	Sir Thomas More's (1478–1535) *History of Richard III* describes how Richard III used his idiosyncrasy to strawberries to undermine his political opponent Lord Hastings
1698	Sir John Floyer describes how ‘no Distemper requires more orderly Diet than Asthma’, advising ‘a very frugal and simple diet’
1773	William Cullen (1710–1790) defines ‘idiosyncrasy’ as ‘a peculiarity of temperament in a particular part of the system’ and associates it with food in *Materia Medica* (1773)
1778	John Fothergill (1712–1780) links migraine or ‘sick head‐ach’ to foods in a 1778 lecture, singling out rich foods in particular
1860	Henry Hyde Salter (1823–1871) lists ‘alimentary irritants (errors in diet)’ as one of the four triggers of asthma in his *On Asthma* (1860)
1902	Charles Richet (1850–1935) coins the term anaphylaxis and links it to food sensitivities
1905	Francis Hare (1858–1928) publishes *The Food Factor in Disease*, which attributes dozens of symptoms to diet
1906	Clemens von Pirquet (1874–1929) defines allergy as ‘any form of altered biological reactivity’
1908	Alfred Schofield describes desensitising a child to egg in *The Lancet*
1931	Albert H Rowe (1889–1970) and son Albert Rowe Jr publish the first food allergy textbook
1939	Helen Morgan publishes the food allergy cookbook *You Cannot Eat That! A Manual and Recipe Book for Those Who Suffer Either Acutely*, *Mildly (And Perhaps Unconsciously) from Food Allergy*
1950	T. Wood Clarke surveys 171 Canadian and American allergists about the relationship between allergy and ‘character problems’ in children. Over half attribute such problems to food
1962	Theron G Randolph (1906–1995) publishes *Human Ecology and Susceptibility to the Chemical Environment*, which blames food chemicals for symptoms of allergy
1965	Teruko Ishizaka (1926–2019) and Kimishige Ishizaka (1925–2018) discover immunoglobulin E (IgE)
1972	A case of fatal peanut anaphylaxis is reported in the American media, one of the first instances of a peanut allergy fatality
1974	Ben F. Feingold (1899–1982) publishes *Why Your Child is Hyperactive*, which blames food additives for hyperactive behaviour in children
1982	Joseph Fries (1902–1982) warns about the potency of peanut allergens in *Annals of Allergy*
1988	Fatal peanut allergy cases are reported in the *Canadian Medical Association Journal* and the *Journal of the American Medical Association*
1991	The predecessor to Food Allergy Research and Education is founded in the US by Anne Muñoz‐Furlong
1994	The Anaphylaxis Campaign is founded in the UK. by David Reading
2005	Sabrina's Law is passed in Ontario, Canada, which requires schools establish and maintain policies to protect anaphylactic students
2006	Peanuts are banned from Edmonton, Alberta's Commonwealth Stadium
2013	President Barack Obama signs Emergency Epinephrin Act
2021	Natasha's Law requires food providers to provide clearer and more complete food labels in the UK

Smith's research also indicates that there was medical and popular interest in food allergies prior to the rise in peanut allergies.^12^ By the 1930s, food allergy textbooks and cookbooks were being published. One indication of a broader awareness of food allergy is demonstrated in a 1933 article about a walrus at a zoo believed to be suffering from food allergies.^13^ After the Second World War, however, debates about the prevalence of food allergy emerge. First, psychosomatic theories of allergy became prominent, prompting some allergists to suggest that symptoms, such as asthma or urticaria, were not caused by allergens, but were psychologically based.^14^ By the 1960s and the publication of Rachel Carson's (1907–1964) Silent Spring (1962), allergists, such as Theron Randolph (1906–1995) and Ben Feingold (1899–1982) were beginning to blame processed foods and food additives for allergic symptoms.^15^, ^16^, ^17^, ^18^, ^19^ There was also Selye's concept of stress, which situated allergy as a disease of adaptation.^20^ The discovery of immunoglobulin E in 1966 by Japanese immunologists Teruko Ishizaka (1926–2019) and Kimishige Ishizaka (1925–2018) did little to resolve these disputes.^2^ Nettleton, Woods, Burrows and Kerr demonstrate that issues related to definition, diagnosis and aetiology continue to fuel disagreements about food allergy.^21^

**FIGURE 3:**
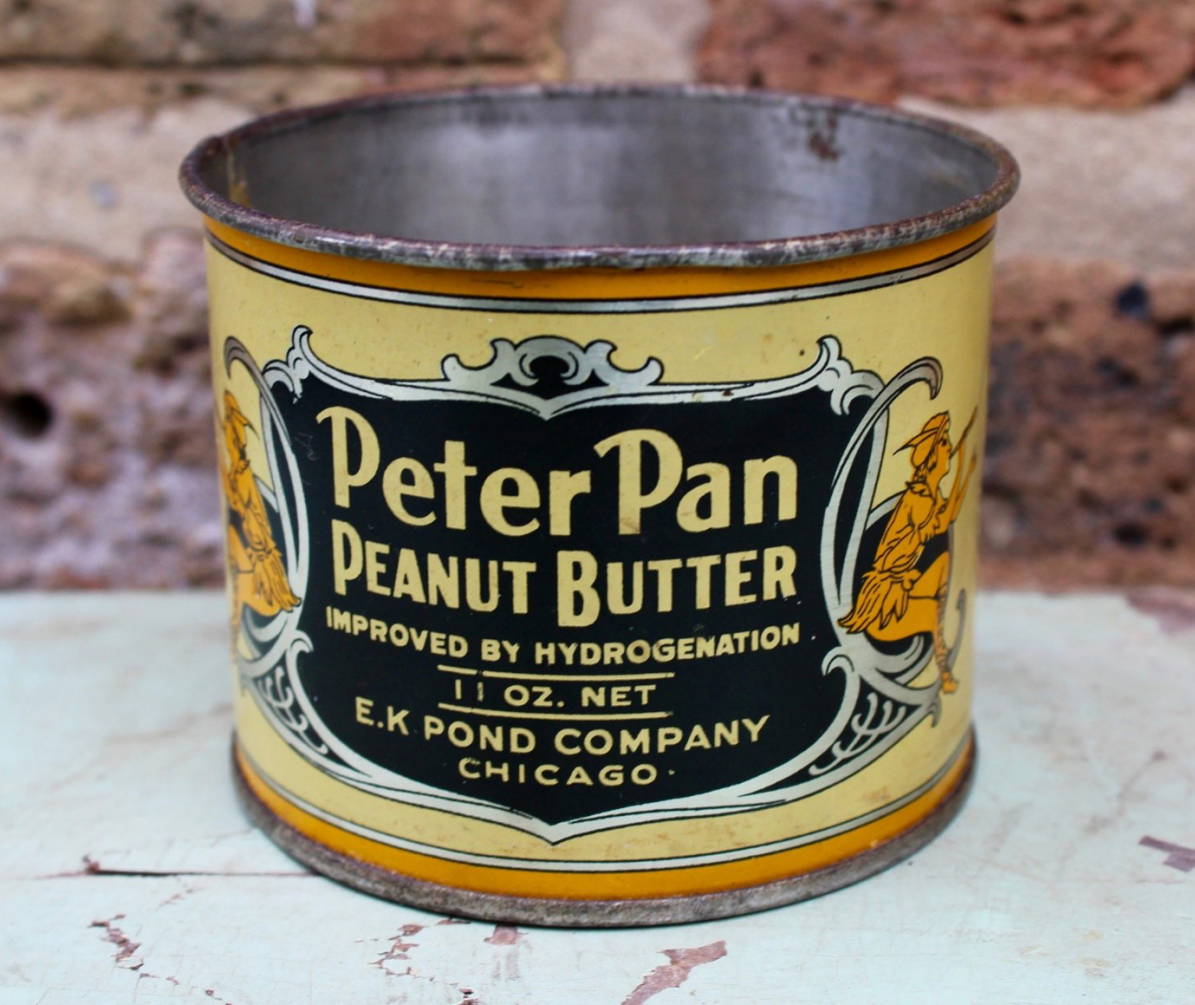
Peanut butter tin from the 1920s. https://www.madeinchicagomuseum.com/single‐post/e‐k‐pond‐peter‐pan/.

Social scientists have also explored the perceived rise in food allergies. In a 1997 paper, Cone and Martin attributed the rise of allergies and autoimmune disorders to changes in the production, transportation and consumption of food triggered by globalisation.^22^ They speculated that, as people increasingly consumed highly processed and chemicalised food that was sourced far from where they lived, their immune systems were more likely to overreact to benign components in food, airborne particles, such as pollen, and even its own tissue. The essay built on Martin's pioneering anthropological research on immunology.^23^, ^24^ In terms of dietary change and the rise of peanut allergy, it is worth noting that peanuts and peanut products were consumed in great numbers long before the increase in peanut allergies during the late 1980s. A song about peanuts ‘Eating Goober Peas’ can be dated to the US Civil War era and peanut butter was invented during the 1890s.^25^ Allergists and immunologists have provided indirect evidence that increased consumption of roasted peanuts in populations might underlie increases in peanut allergy.^26^ However, research from Montreal, Canada during the 2000s also provided evidence that the rate of peanut allergy was plateauing in that city among young children, making questions about its epidemiology more complicated (Figure 3).^27^

Anthropologists have also tackled the hygiene hypothesis. Some have readily accepted the theory, even suggesting that adding more ‘dirt’ to our lives could reduce certain autoimmune diseases or exploring the hypothesis' social implications.^28^ Clough, for instance, argues that girls in industrialised countries are expected to be cleaner than boys, meaning that they are exposed to fewer environmental microbes.^29^ If the hygiene hypothesis is valid, she continues, this may explain higher rates of allergy, asthma, autoimmune disease and depression in women. Others have revealed the class dynamics of the hygiene hypothesis. Focussing on Canada, Minaker, Elliott and Clarke found that some allergists believed that people from wealthier backgrounds were more likely to have food allergies because they were thought to live in more hygienic environments (interestingly, the low‐income people in their study blamed allergies on food additives, vaccinations and breastfeeding practices).^30^ The corollary is that poorer people must live in dirtier environments.^31^ Such classist assumptions may influence how clinicians think about the degree to which low‐income allergic individuals will adhere to advice about avoiding allergens. This is despite the fact that poorer people often face more risks (real or imagined) from their allergies because they may have less access to epinephrine injectors, may not be able to afford hypoallergenic products and may have to access facilities, such as food banks, where there is a perceived risk of cross‐contamination.^29^ Another problem is conflicting evidence from the US about the relationship between socioeconomic status and food allergy.^32^, ^33^ Higher levels of food allergy, however, have been consistently associated with specific racial groups, for example Black Americans in the United States.^34^, ^35^, ^36^

Atiim et al. also explored how the ‘political ecology of health … which explicitly examines the interactions between biology, broader sociopolitical and environmental processes’ affects experiences and understandings of allergy in Ghana.^37^ Their interviews revealed that factors ranging from inadequate access to healthcare to scepticism about food allergy accentuate the risk faced by allergic individuals. By emphasising the increase of food allergies in Ghana, this particular study also raises questions about the hygiene hypothesis, which posits that rates of allergy should be lower in low‐ and middle‐income countries. Studies have suggested significant global variation in food allergies between rural and urban populations, but the authors of the research on Ghana suggest that such variation might be narrowing.^38^, ^39^, ^40^

Other social scientists, however, have unpacked the hygiene hypothesis. Noting dissatisfaction with the term, Blackwell emphasises how the relationship between the immune system and the external environment should be characterised by plasticity and how multiple factors, including nutrition, may have affected this relationship.^41^ Similarly, Haeusermann, Waggoner and others have provided sociological explanations for the apparent rise in food allergies, emphasising, for instance, how the peanut allergy epidemic was co‐constructed by a wide range of stakeholders with differing agendas.^42^, ^43^, ^44^ These different interpretations mirror differences in how allergic disease has been approached by historians. While Mitman and Murphy start with the presumption that environmental factors, especially airborne chemicals, contribute to allergies and asthma, Jackson has been more circumspect, subjecting environmental theories of allergy to the same degree of scrutiny as any other explanation.^5^, ^45^, ^46^

## 3 CONSTRUCTING THE RISK OF FOOD ALLERGY

Public health policy regarding food allergy has relied in part on epidemiological evidence, but the dramatic narratives surrounding food allergy have also played their part.^2^, ^47^; Social scientists exploring preventive approaches to food allergy have often used the concept of ‘risk society’ to frame their analysis.^48^, ^49^ Beck defines a risk society as those that develop ‘systematic way[s] of dealing with hazards and insecurities induced and introduced by modernisation itself’.^49^ Such a definition helps to explain how societies have dealt with food allergy. Food allergies have been associated with modernity and, while they themselves represent hazards, they have also bred anxieties not only about the direct threat they pose but also what they represent more generally.^5^ To many they are a good example of what Rosenberg has called a ‘disease of civilization’.^50^

The mitigation of food allergy risks has taken a variety of forms, including the banning of potent allergens, especially peanuts, from public spaces, better labelling of ingredients and mandating the availability of epinephrine in schools.^51^ In turn, these initiatives have varying effects on the non‐allergic public. The majority of people, for example, could benefit from clearer and more comprehensive ingredient labelling. While stocking backup epinephrine might be a cost shared by all, it does not limit anyone's rights or freedoms, though, as Glabau argues, it is important to consider what epinephrine injectors signify in moral, economic and biomedical terms.^52^ In contrast, banning peanuts and tree nuts, does restrict access to food sources that are otherwise highly nutritious. Moreover, it may stoke anxieties about food allergies that could be misplaced.^51^ Finally, as Rous and Hunt have argued, the emphasis on the immediate risks posed by allergens in schools has undermined health promotion strategies aimed at encouraging healthier eating which emphasises what to eat, rather than what to avoid.^47^ This is particularly problematic in the case of peanuts and tree nuts, which are highly nutritious.

Social scientists also show that risk is understood subjectively by individuals. Peanut allergy, for example, has been described by DeSoucey and Waggoner as existing ‘in the interstices of scientific facts, commercial interests, and shifting cultural tenets’. In turn, the risk posed by peanuts ‘is a rich manifestation of disputes over the boundaries of responsibility for health and safety for self and others’.^53^ Moreover, Cook has argued that allergic individuals do not perceive these risks on their own.^54^ Instead, their perception is entangled in their response to particular physical and social environments, including the influence of other people.^55^ An individual, for instance, might be more willing to poke around and sniff a take‐away meal consumed at home, than they would in a busy restaurant or at a dinner party. The way young people with food allergies perceive risk may be particularly fluid. Adolescents still rely on their parents to decide what is ‘safe’ for them in certain spaces, but they may simultaneously find themselves in situations where they have to determine the degree of risk themselves. Making this dynamic more complex are issues such as peer pressure and the fact that risk‐taking is a facet of life for most adolescents.^56^

Nor is risk always constructed by allergic people straightforwardly. As Hu, Kerridge and Kemp have argued, ‘rational descriptions of risk that exclude qualitative and emotion based evaluations will only partially explain the experience of risk, and the responsibility for risk’.^57^ Gaivoronskaia and Hvinden's survey of people in Norway, for instance, found that those with food allergies were more likely to associate food with risk than those without food allergies. This finding may not be surprising, but the difference between the two groups (66% for the allergic group and 52% for the non‐allergic group) was not as great as one might think.^58^ Similarly, in a Netherlands study both allergic and non‐allergic people were equally confused regarding the level of risk associated with specific precautionary labels present on foods.^59^ Complicating the matter further are stories about particularly bizarre reactions to allergens, such as those apparently triggered by kissing another person or airborne allergens.^60^, ^61^ Such reactions may be breathtakingly rare and difficult to attribute, but—as with concerns about the exchange of bodily fluids during the early years of HIV/AIDS—they captivate us and the media,^62^ and influence how individuals and societies calculate risk.^23^, ^60^

The ambiguities and uncertainties that Nairn has identified with immunotherapy for life‐threatening food allergies also show how the perception of risk is contingent on numerous factors.^63^, ^64^ Immunotherapy for food allergy can be traced to 1908, just 2 years after von Pirquet coined the term allergy, but due to the risks involved it fell out of favour.^65^ In recent decades, however, it has been hailed as a ‘paradigm shift’ in allergy treatment.^63^, ^64^, ^65^ As Nairn has shown, however, variations in immunotherapy products and practices, and differing claims about what constitutes the ‘best’ ways to do immunotherapy—‘standardised’ FDA regulatory approved or in a ‘real food’ format—mean that allergic patients still have to contend with scientific and medical uncertainties.^63^, ^66^ This is not the least because external factors, such as stress, have historically been thought to exacerbate the severity of allergic responses, a factor under consideration again today.^67^, ^68^ In turn, scholars have also highlighted that psychosocial stress is another risk that people dealing with allergies face. The healthy development of children with food allergies is thought by some to be impeded by social isolation (e.g., from being excluded from activities, such as birthday parties) and anxieties about accidental exposure.^69^ The food worries of both children and adults with food allergies have also been linked to increased levels of loneliness.^70^ Moreover, Rocheleau and Rocheleau have found that not only are children with food allergies more likely to be bullied, but that being the parent of an allergic child who is bullied also leads to greater stress, anxiety and depression.^71^

Most social scientists have focussed on how the risks and uncertainties surrounding food allergy have been constructed and understood. In so doing, many scholars have relied on theories of governance, arguing that, despite the lack of incontrovertible scientific evidence concerning the epidemiology and aetiology of food allergies (as well as the effectiveness of public health responses), some form of response in the form of is expected. They emphasise that, in order to understand such responses, it is crucial to acknowledge the context in which such responses occur.^72^ It is also important, however, for social science and humanities scholars to be self‐reflective in terms of how they themselves assess the construction of such risks and uncertainties. Some scholars have questioned the risks surrounding food allergies. Others focus more on how the risks posed by food allergies are experienced by those dealing with food allergies as either sufferers or parents. In order to explain this difference, it is worthwhile to consider the methodology of such scholars. Those who interview food allergy sufferers and their parents appear more sympathetic to how they interpret the risk posed by food allergy. Indeed, some such scholars suffer from food allergies themselves or are the parents of sufferers.^60^ The final section of the article, therefore, turns more in depth to approaches that attempt to capture the experiences of those coping with food allergies.

## 4 EXPERIENCING FOOD ALLERGIES

Humanities and social science scholars can contribute to our understanding of food allergy by studying how food allergy is experienced by those affected by it. Methods ranging from oral history interviewing and participatory action research to online surveys and focus groups all tap into what it is like to experience food allergies. While at times they reveal what we might suspect already, for instance, that mothers bear the burden when it comes to supporting their allergic children, at other times the insights are surprising. For example, people with food intolerances find their condition more ‘socially problematic’ than those with severe anaphylactic allergies.^73^ People dealing with food allergies also have a valuable type of expertise that can help inform understanding of food allergy.^73^, ^74^

One example of this is the story of the Feingold diet, a food additive‐free approach to Attention Deficit Hyperactivity Disorder (ADHD). Developed in the early 1970s, when concerns about food additives were mounting, the diet, designed by respected allergist Ben Feingold, divided opinion.^19^ Clinical trials were conducted to test the diet, but close inspection of these revealed significant flaws and/or bias among both those that supported and discredited the Feingold diet. After Feingold died in 1982, medical and media interests waned. But parents who found the diet to be effective continued to promote it. Smith's oral histories of these parents, the findings from which resemble Swedish research on the pathways parents take when they suspect their children are allergic,^74^ shows how their experiences and insights not only helped others struggling to help their children but also reinvigorated the research agenda. By the early 2000s, public pressure on the Food Standards Agency in the UK resulted in new studies. This time, the studies were better designed and, though they did not unequivocally support the Feingold diet, they did indicate a link between synthetic food chemicals and hyperactive behaviour and a specific genetic vulnerability to this in some children.^75^, ^76^ Soon the European Food Standards Agency mandated warning labels on products containing certain food dyes, the UK National Health Service was publishing guidance to the public about the link between additives and hyperactivity and food companies, such as Marks and Spencer's, were voluntarily removing such dyes from their products.

A recent book by Glabau delves even deeper into the experiences of those affected by food allergies, providing an in‐depth ethnography of an allergy clinic as well as 70 interviews of people involved in food allergy advocacy.^77^ Her research highlights how racism, neoliberalism and sexism have constrained the efforts of food allergy advocates in trying to create safer spaces for the allergic person. Analysing parenting magazines, Muñoz and Quirke have also emphasised how coping with food allergies is gendered, with mothers expected to do the majority of ‘foodwork’ and ‘emotion work’ related to preventing accidental exposure.^78^ Mothers are also more likely to be tasked with liaising with schools and ensuring that a prevention plan is in place.^79^ The rich, descriptive style employed by humanities and social science scholars can illustrate these experiences and emotions effectively. Cook's article on navigating food allergies in Japan, for instance, begins with a description of how the appearance of an ice cream van in a park on a hot summer day is a source of tension, not glee, for a 10‐year‐old with milk allergy.^80^ Such a depiction helps the reader empathise more with those dealing with food allergies and stresses the role of fear (whether rational or not) in how people understand and experience food allergies.

## 5 CONCLUSION

Writing in 2015, Page‐Reeves noted that food allergy had not been adequately addressed by social scientists.^81^ Eight years later, both humanities and social science scholars are increasingly studying food allergy, but significant gaps remain. Arguably the most important is that social scientists, humanities scholars and food allergy researchers still research and publish in silos. More genuine interdisciplinarity would be welcome. A model for this might be the social psychiatry research that blossomed in the 1950s in the United States, which provided a solid foundation for the epidemiology of mental illness.^82^ Social psychiatry research paired psychiatrists with social scientists, resulting in key insights about mental health. Could the same be done for food allergy?

Some social sciences and humanities research on food allergy also has a tendency to make assumptions about the root causes, prevalence and impact of food allergy. Concepts, such as the hygiene hypothesis, for instance, are either accepted as read or downplayed without sufficient evidence. Another way for social scientists and humanities scholars to contribute to these debates is to employ the approach Jackson took with his now‐classic history of allergy, that is, subjecting both sides of the debate to rigorous critique and analysis. Doing so may lead to a clear answer or confirm the need for nuanced interpretation.

Finally, scholars should accelerate ongoing work that articulates the experiences and understandings of those dealing with food allergies as patients, parents, advocates, scientists and clinicians. The voices of children and adolescents, in particular, have tended to be overshadowed in current research, which is an oversight. There is also a need for qualitative longitudinal studies that bring together the understandings and experiences of mothers who have raised children with food allergies to adulthood. This is particularly important given the changing advice related to infant feeding and the timing of allergenic food introduction.^83^ More generally, a larger body of qualitative evidence, provided, for example, by large‐scale oral histories, could be crucial in answering questions about the aetiology and epidemiology of food allergy and the effectiveness of clinical practice and public health policy. All of this, however, relies on the understanding that the insights from researchers from many disciplines can contribute to our knowledge of food allergy and deserve to be respected. [Correction added on 28 June 2023, after first online publication: Some in‐text reference citations on pages 5 to 7 have been updated].

## AUTHOR CONTRIBUTIONS

The manuscript was written and prepared by MS. Figures and tables were prepared by MS. MS has reviewed and approved the final submitted manuscript.

## CONFLICT OF INTEREST STATEMENT

The author has no conflicts of interest to declare.

## Data Availability

This is a review article and is solely based on published material.
